# Supplementary material for: Fc-engineered broadly neutralizing antibodies overcome cytokine-induced CD16 cleavage to drive potent ADCC against diverse HIV-1 strains
Source: mBio. 2026 Aug 21;17(9):e01724-26. doi: 10.1128/mbio.01724-26 (PMC13556199; doi:10.1128/mbio.01724-26)
Supplement: Supplementary file 1 [file mbio.01724-26-s0002.pdf]

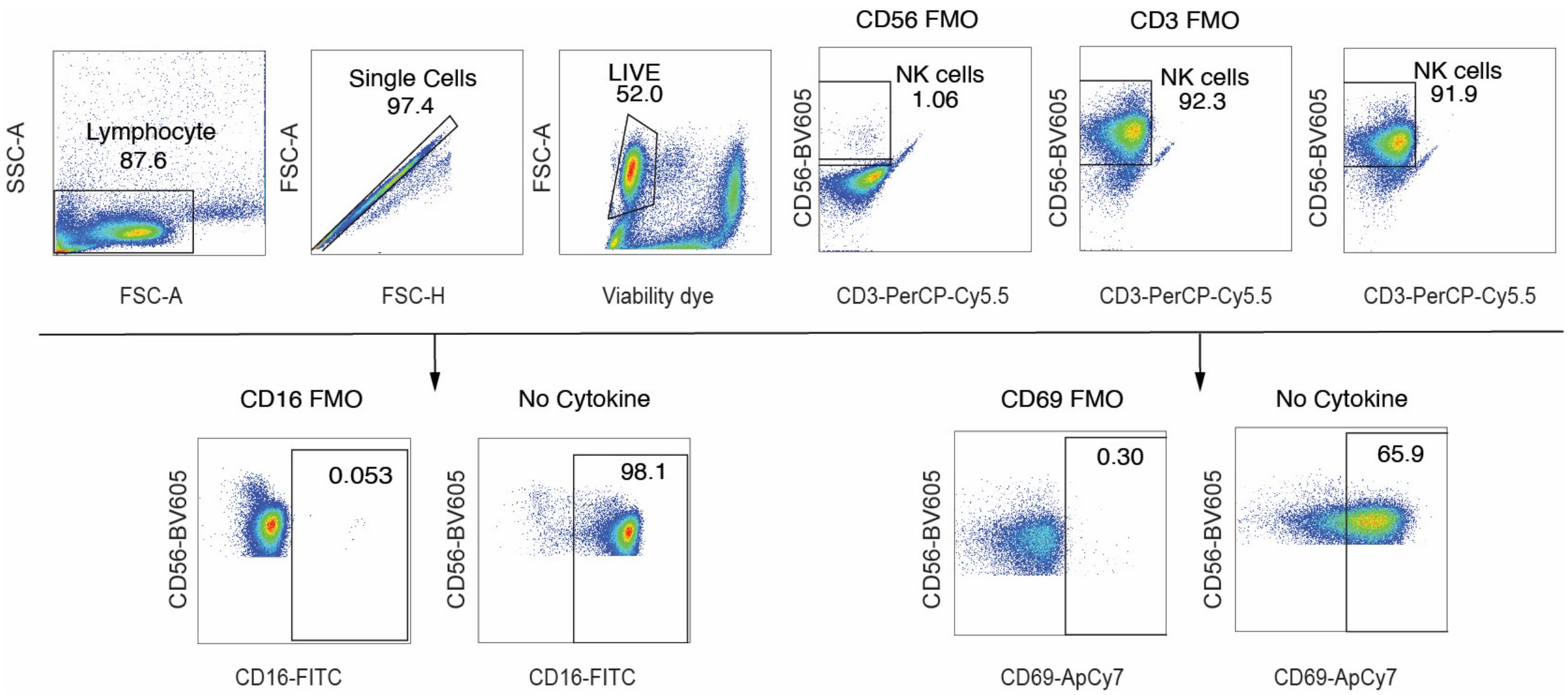

Fig. S1. CD69 gating strategy. Cells were gated first based on lymphocytes followed by single cells then live cells. NK cells were then separated based on the CD3 and CD56 expression. CD16 and CD69 were measured on CD56+CD3- NK cells.

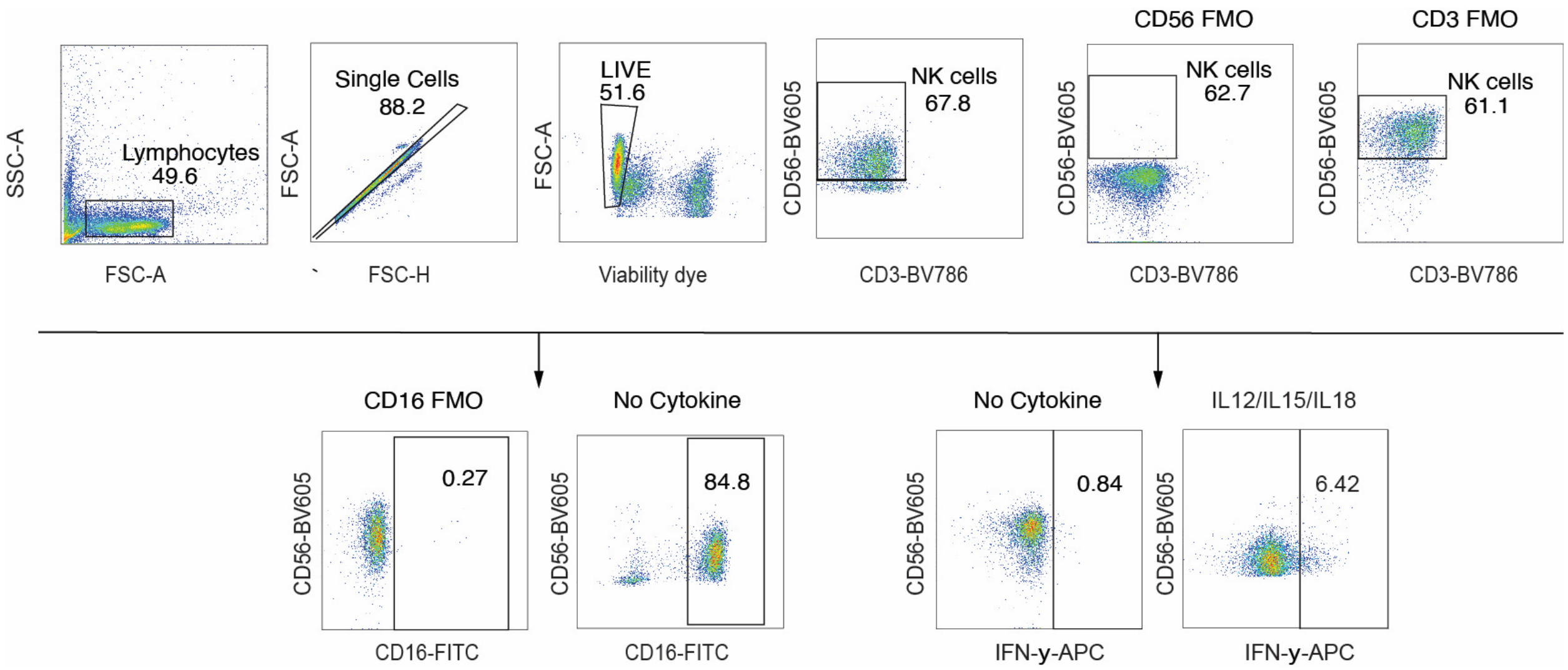

Fig. S2. IFN-γ gating strategy. Cells were gated first based on lymphocytes followed by single cells then live cells. NK cells were then separated based on the CD3 and CD56 expression. Following the CD16 gMFI values were measured on NK cells. CD16 and IFN-γ were measured on CD56+CD3- NK cells.

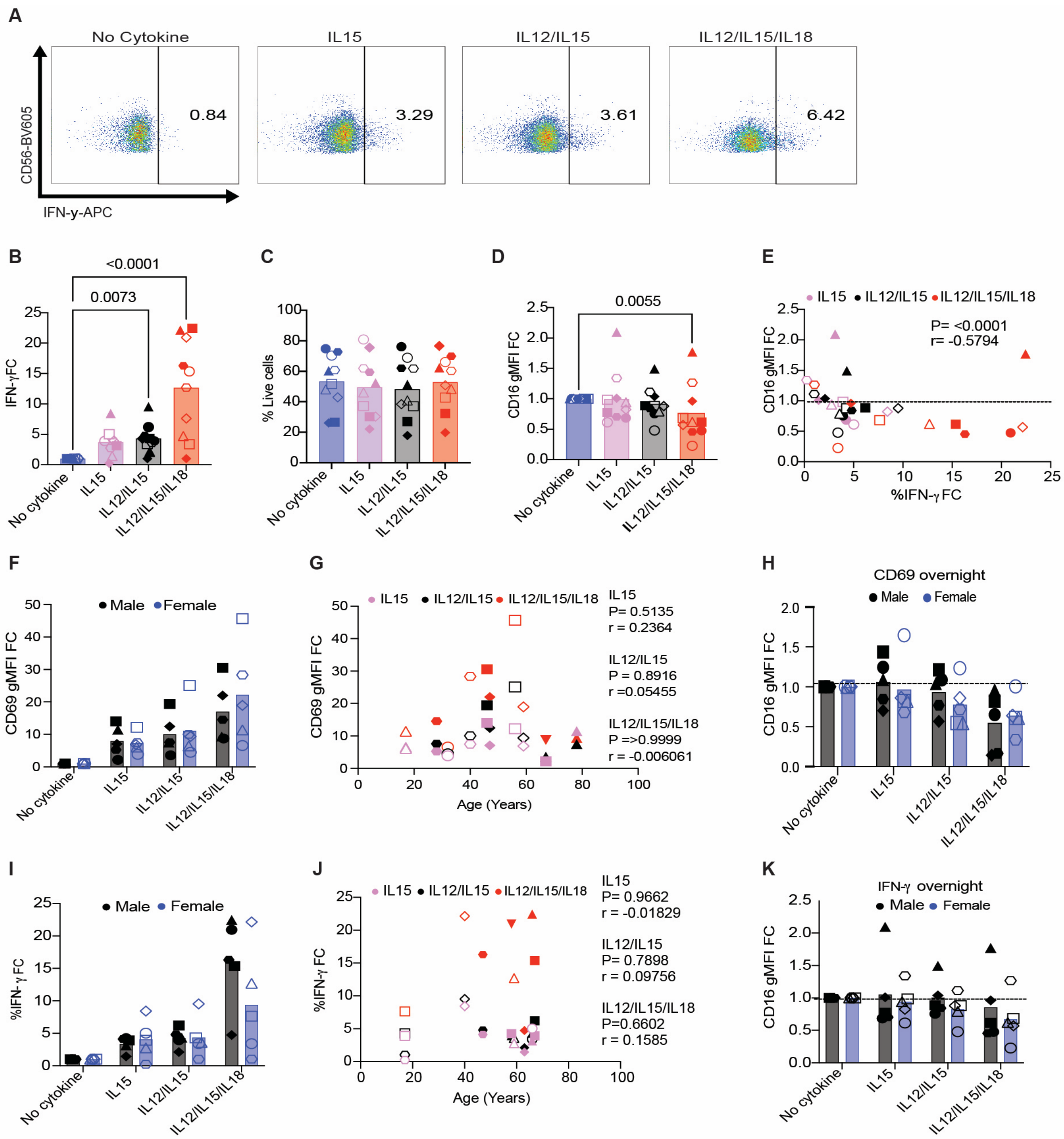

Fig. S3. Cytokine stimulation enhances NK cell activation while downregulating CD16 expression through ADAM17-dependent shedding. (**A**) Representative flow plots of IFN-γ induction of NK cells stimulated with IL-15, IL-12/IL-15, or IL-12/IL-15/IL-18. (**B**) IFN-γ induction across donors (n=10). (**C**) Viability upon different treatments. (**D**) Changes in CD16 expression across multiple donors upon cytokine treatment. (**E**) Spearman correlation analysis between CD16 expression and IFN-γ induction. (**F**) CD69 induction stratified by biological sex. (**G**) Spearman correlation

between CD69 induction and age. (**H**) CD16 expression upon cytokine treatment stratified by biological sex. (**I**) IFN-γ stratified by biological sex. (**J**) Spearman correlation between IFN-γ induction and age. (**K**) CD16 expression upon cytokine treatment stratified by biological sex. Statistical significance was determine using Friedman test with multiple comparisons compared to No cytokine for panels (**B-D**), and multiple unpaired t tests for panel (**F**, **H**, **I** and **K**). Open symbols represent female donors and closed symbols male donors.

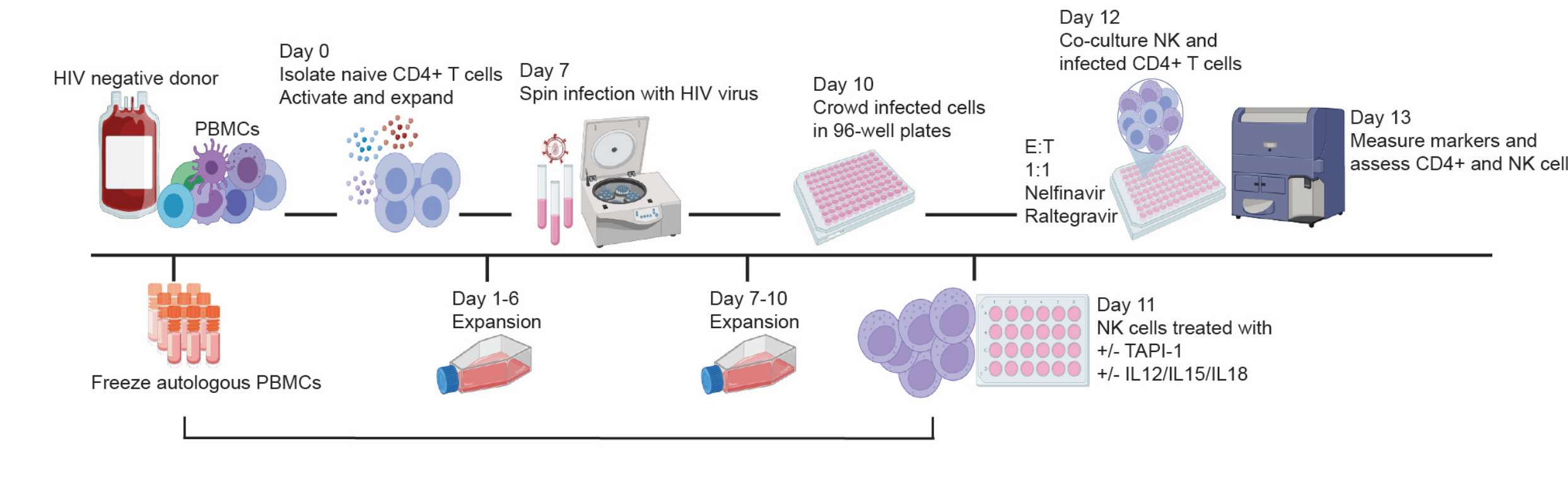

Fig. S4. Experimental setup to evaluate NK natural cytotoxicity and ADCC of autologous HIV-infected CD4T cells.

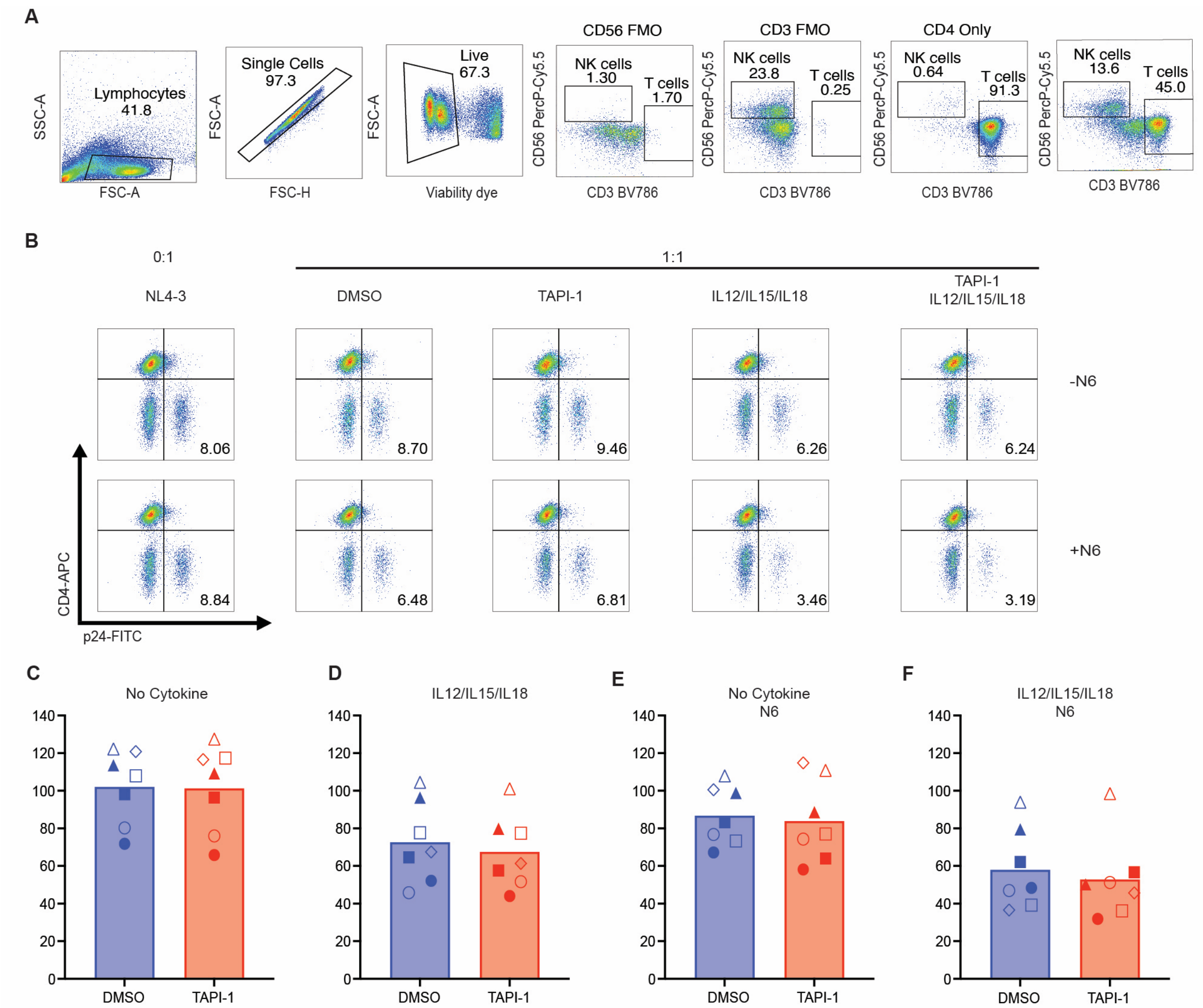

Fig. S5. Inhibition of ADAM17 by TAPI-I does not rescue ADCC. (**A**) Co-culture gating strategy. (**B**) Representative gating strategy to evaluate cytotoxicity. (**C**) Percentage remaining p24+ cells upon coculture of HIV-infected CD4T cells with autologous NK cells comparing DMSO versus TAPI-1 for (**C**) No cytokine pre-treatment, (**D**) IL12/IL15/IL18 pre-treated NK cells, (**E**) No cytokine pre-treatment in the presence of N6, (**F**) IL12/IL15/IL18 pre-treated NK cells in the presence of N6 (n=7). Statistical significance was determined using a paired t-test. Open symbols represent female donors and closed symbols male donors.

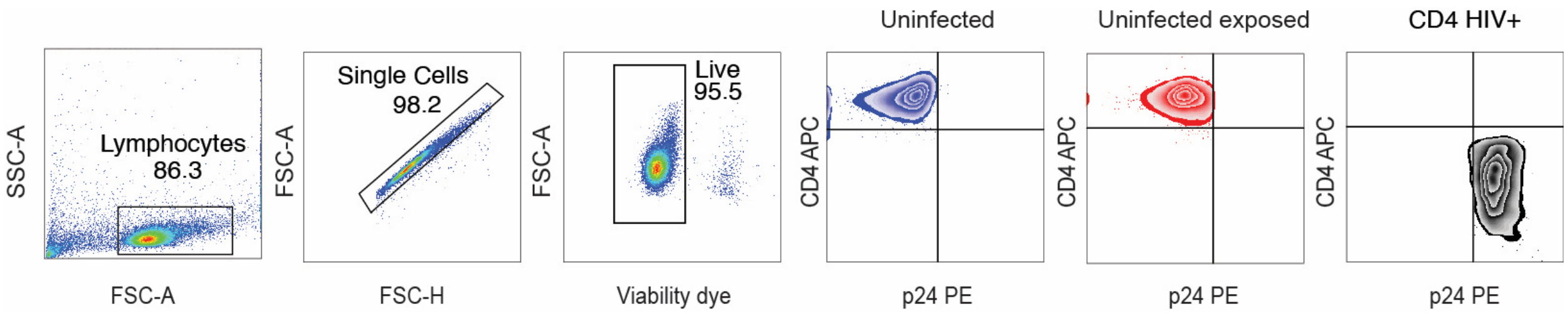

Fig. S6. Gating strategy for Envelope staining for Fig. 2P.

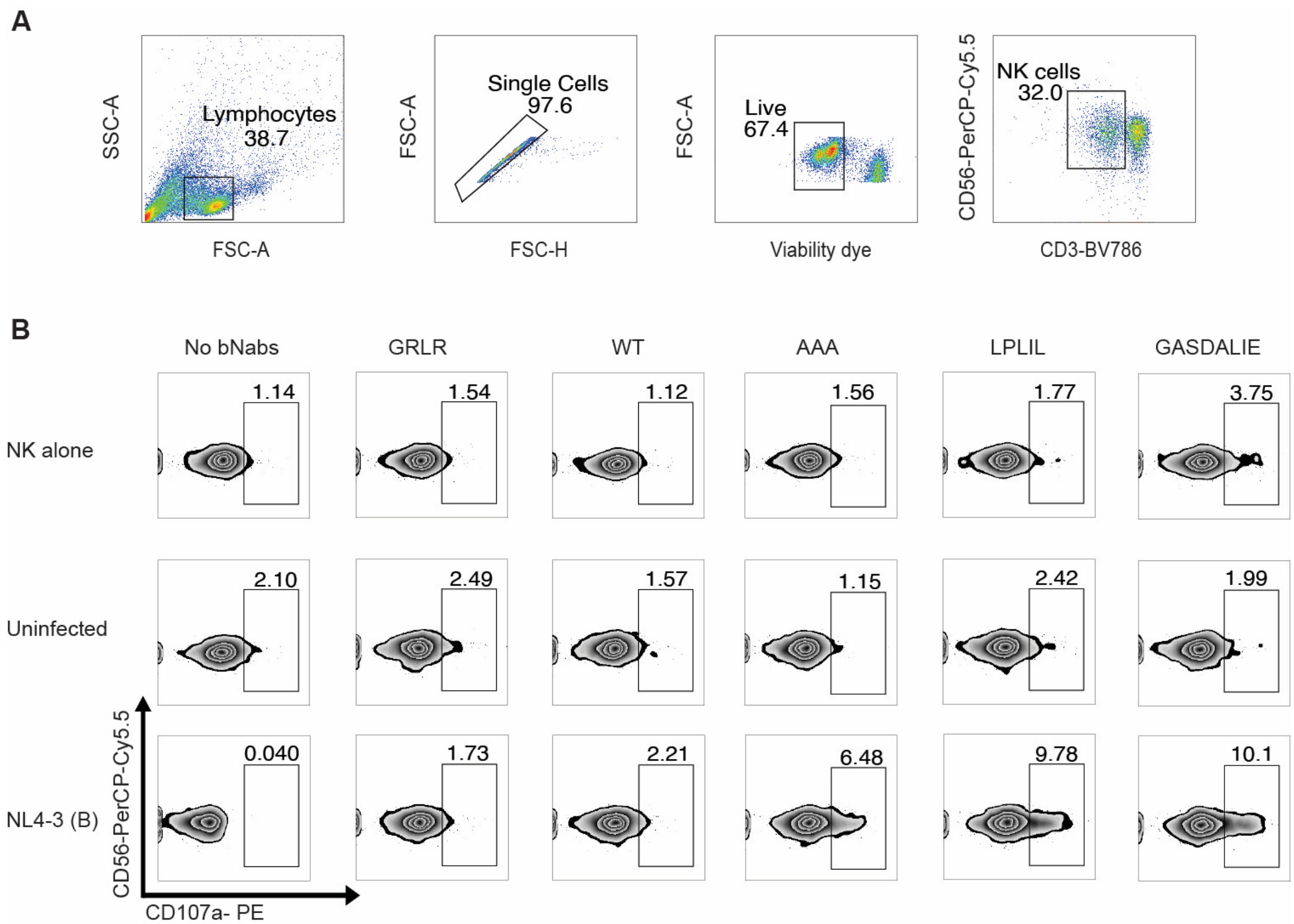

Fig. S7. Supplementary Gating Strategy and Representative Flow Cytometry Analysis of NK Cell Degranulation. **(A).** Gating strategy used to assess NK cell degranulation measured by CD107a. **(B).** Representative flow cytometry plots showing degranulation for NK cell alone or co-culture with uninfected or NL4-3 infected CD4+ T cells in the absence or presence of bNAbs for Fig. 2Q.

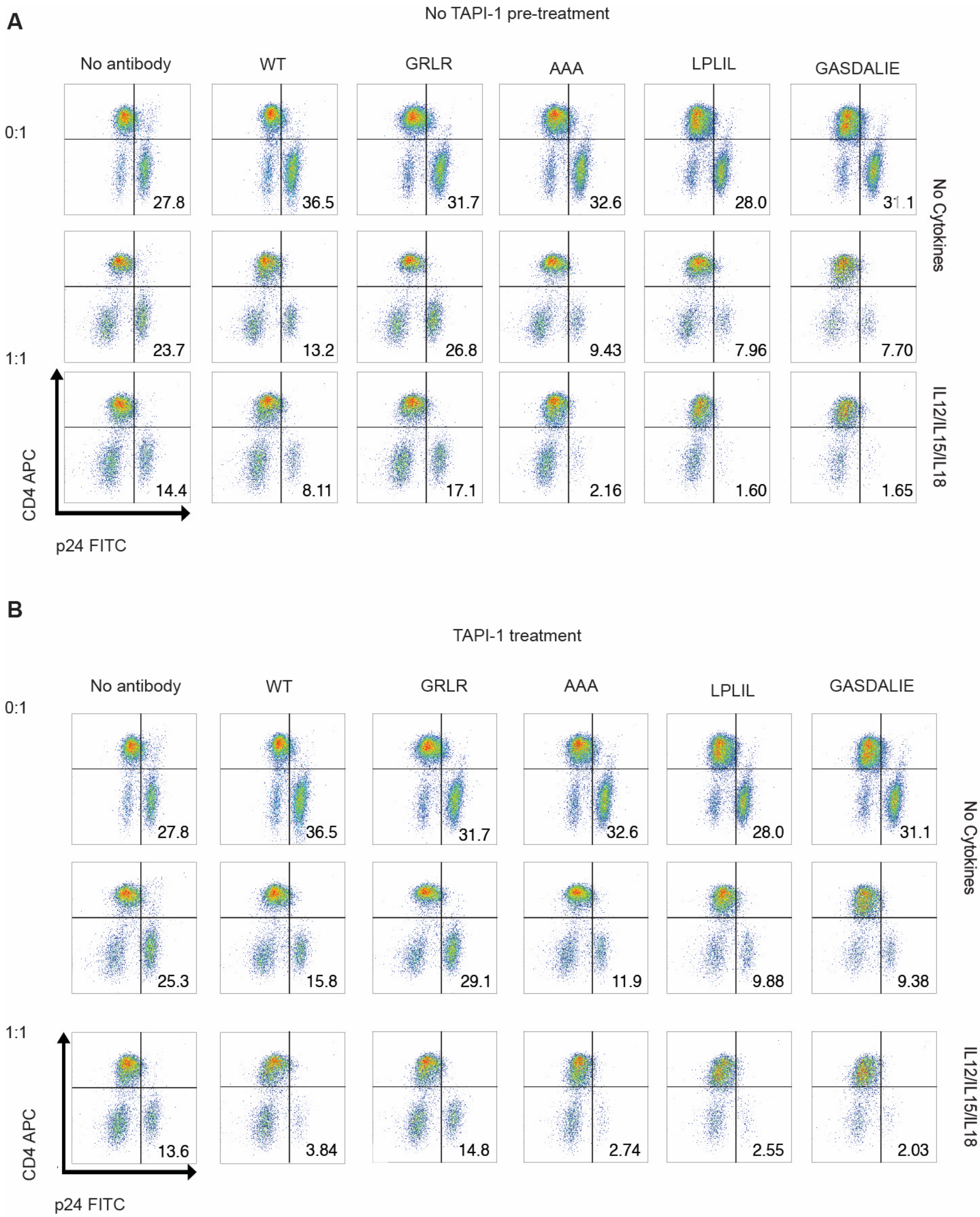

Fig. S8. Representative gating strategy for co-culture experiments in Fig. 3. In the (**A**) absence or (**B**) presence of TAPI-I.

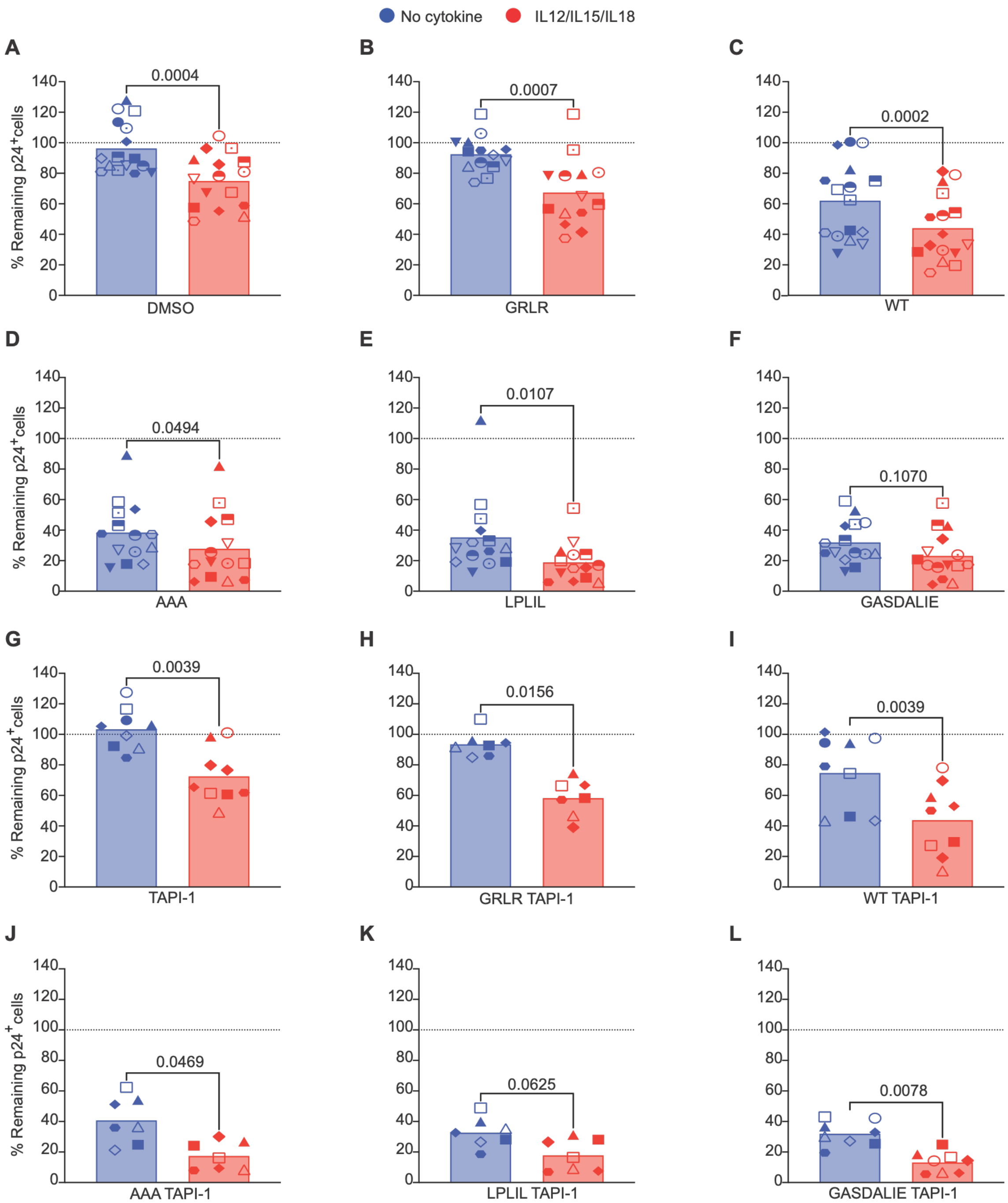

Fig. S9. Comparison of the percent remaining p24+ cells between no treatment control and triple cytokine treatment for experiments shown in Fig. 3. (**A**) DMSO control. (**B**) GRLR. (**C**) VRC07-523-LS (WT). (**D**) AAA. (**E**) LPLIL. (**F**) GASDALIE. (**G**) TAPI-1. (**H**) VRC07-523-LS (WT) and TAPI-1. (**I**) GRLR and TAPI-1. (**J**) AAA and TAPI-1. (**K**) LPLIL and TAPI-1 and (**M**) GASDALIE and TAPI-

1. (n=9-16). Statistical significance was determine using the Wilcoxon test. Open symbols represent female donors and closed symbols male donors.

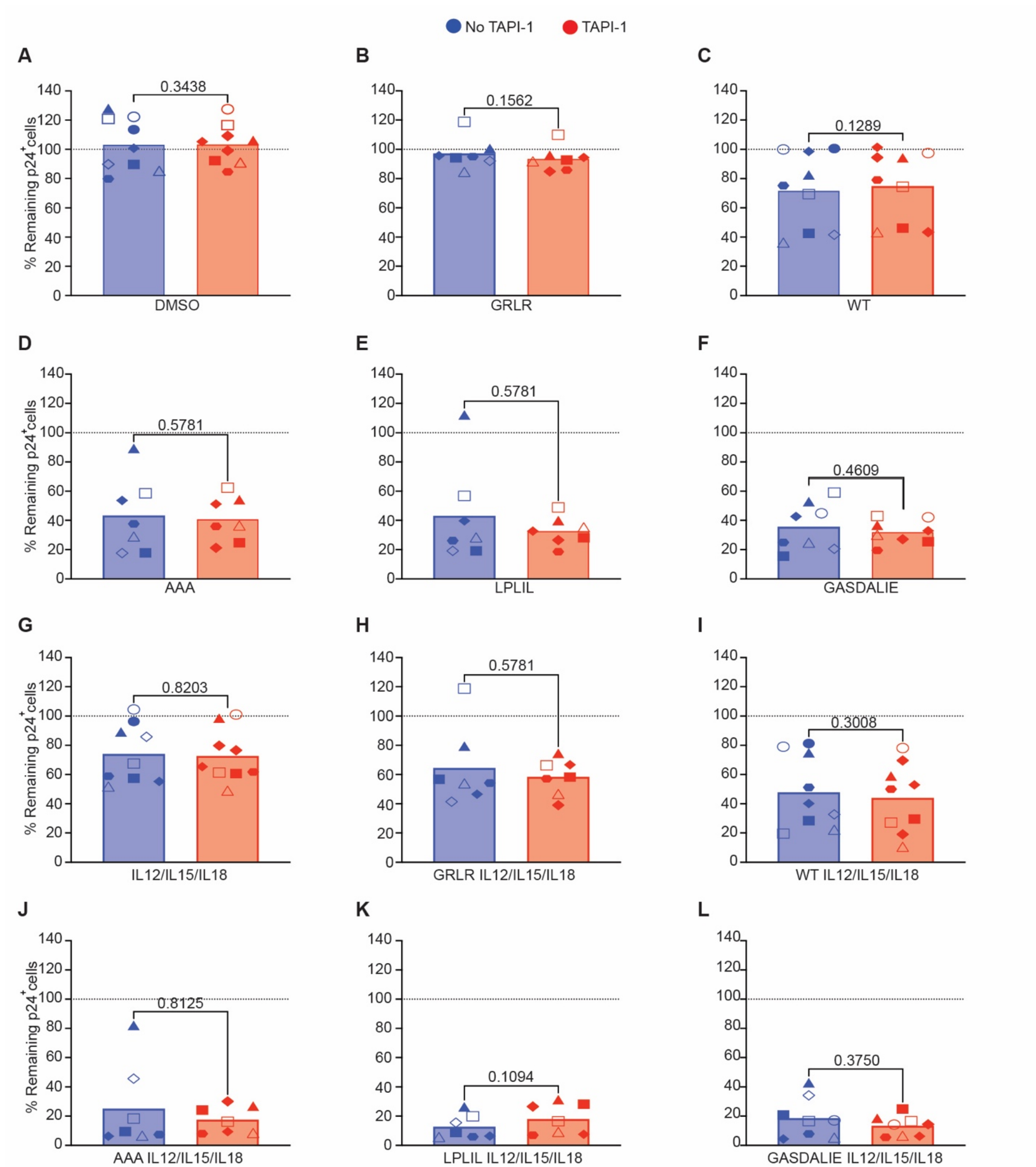

Fig. S10. Comparison of the percent remaining p24+ cells between No TAPI-1 control and TAPI-1 treatment for experiments shown in Fig. 3. (**A**) DMSO control. (**B**) GRLR. (**C**) WT. (**D**) AAA. (**E**) LPLIL. (**F**) GASDALIE. (**G**) IL12/IL15/IL18. (**H**) IL12/IL15/IL18 and GRLR. (**I**) IL12/IL15/IL18 and WT. (**J**) IL12/IL15/IL18 and AAA. (**K**) IL12/IL15/IL18 and LPLIL and (**M**) IL12/IL15/IL18 and

GASDALIE (n=9). Statistical significance was assessed using the Wilcoxon test. Open symbols represent female donors and closed symbols male donors.

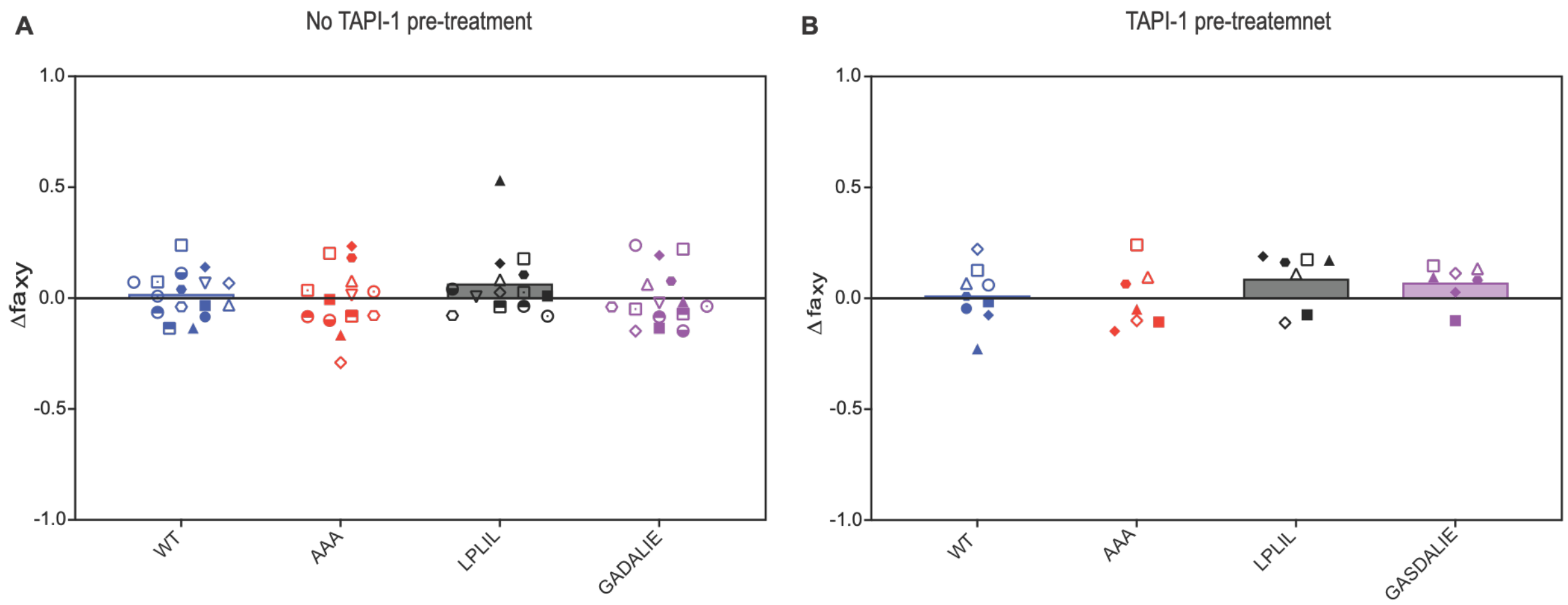

Fig. S11. Synergy evaluation between cytokine treatment and the different Fc-optimized antibodies for experiments shown in Fig. 3. Statistical significance was determined using a one-sample t-test and the Wilcoxon signed-rank test, with a hypothetical value of 0.

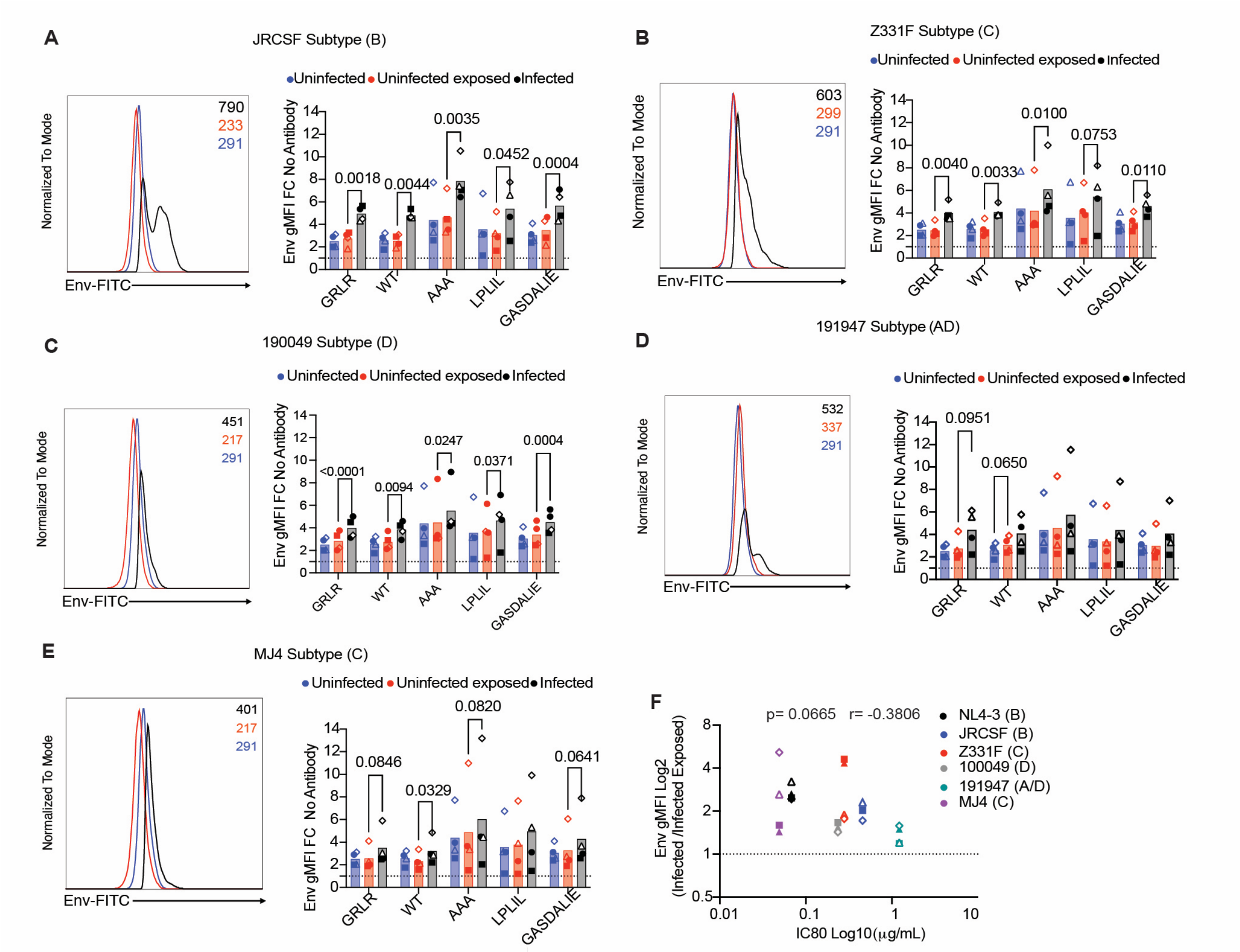

Fig.S12. Binding of Fc-optimized VRC07-523LS mutants across HIV-1 subtypes. (**A**) Spearman correlation between Env binding and neutralization IC80 values. (**B-F**) Representative flow cytometry histograms show antibody binding to Env on HIV-infected cells for the WT antibody in uninfected (blue), uninfected exposed (red), and infected (black). Quantification of Env binding on infected CD4$^+$ T cells was performed in the presence of each antibody variant for JRCSF (**B**), Z331F (**C**), 190049 (**D**), 191947 (**E**) and MJ4 (**F**). (n=3-4). Statistical significance was determined using the two-way ANOVA with multiple comparison to uninfected exposed. Open symbols represent female donors and closed symbols male donors.

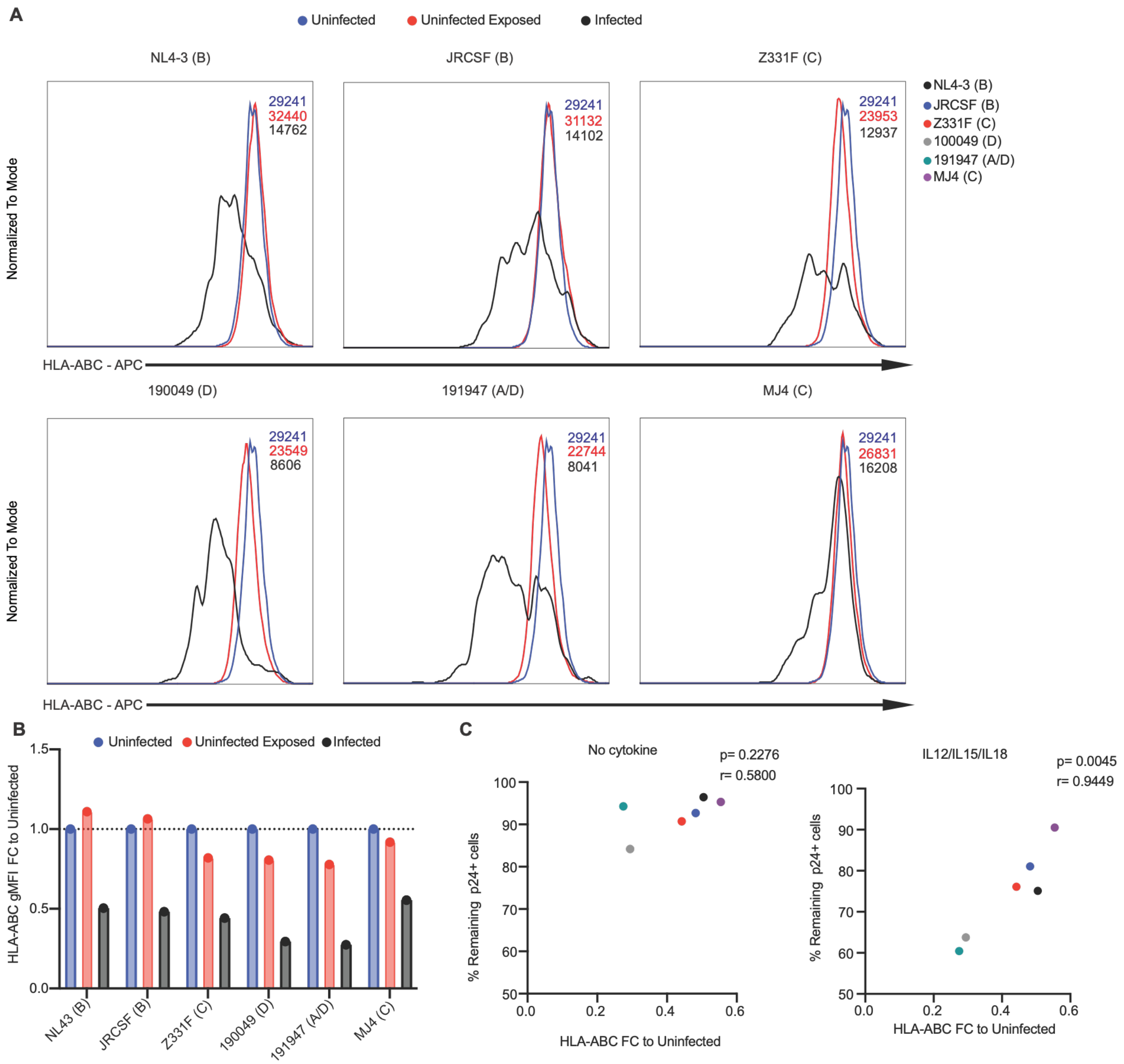

Fig. S13. HLA-ABC downregulation across HIV-1 subtypes used. (**A**) Representative flow cytometry histograms showing HLA-ABC expression on HIV-infected cells in uninfected (blue), uninfected exposed (red), and infected (black). (**B**) Bar graph comparing the HLA-ABC on infected CD4$^+$ T cells among viral subtypes (n=1). (**C**). Spearman correlation between the average of the percent of remaining p24+ cells (no antibody) under no cytokine and IL12/IL15/IL18 condition and HLA-ABC gMFI.

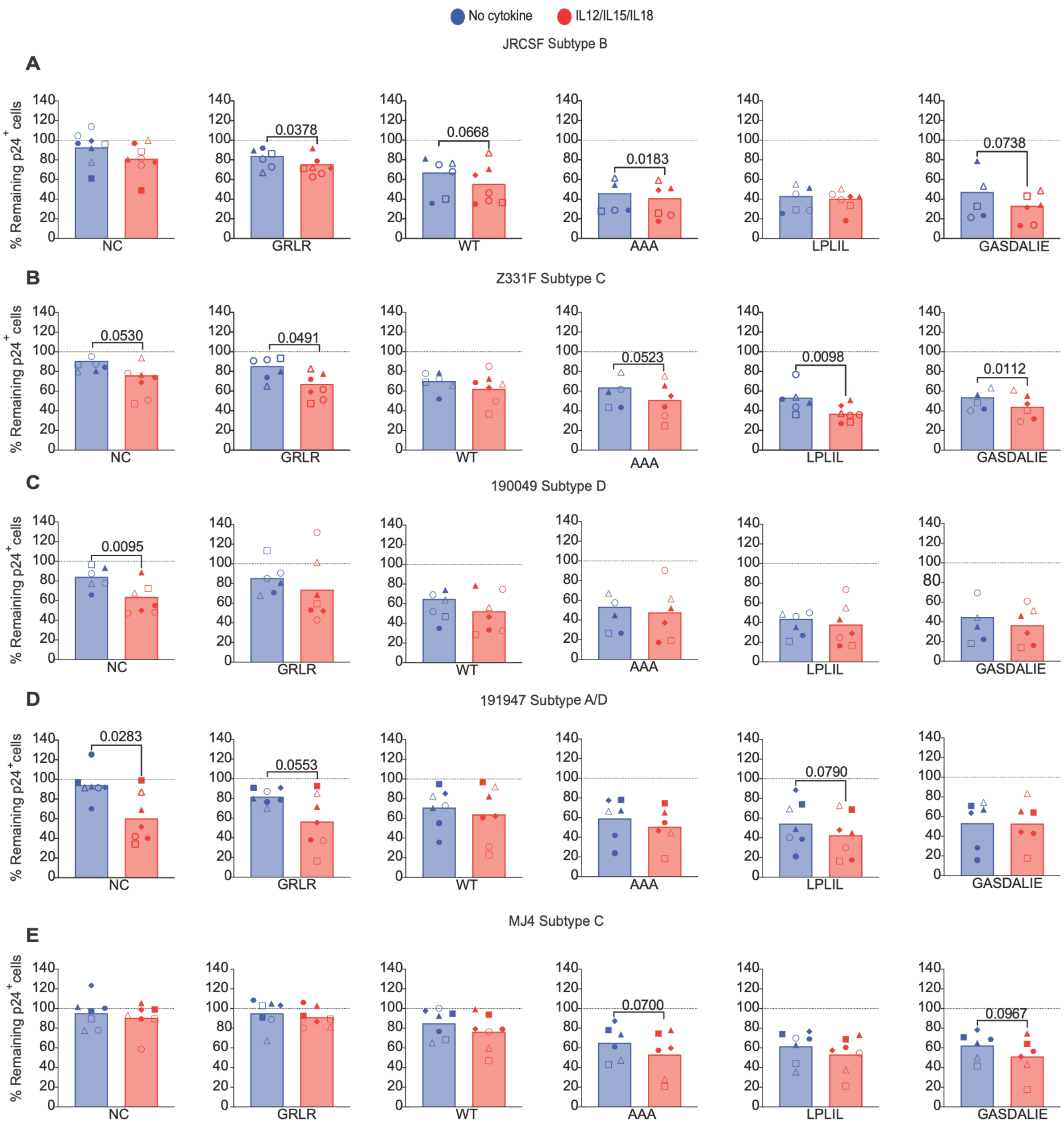

Fig. S14. Comparison of the percent remaining p24+ cells between no treatment control and triple cytokine treatment for experiments shown in Fig. 4. (**A**). JRCSF, (**B**) Z331F, (**C**) 190049, (**D**) 191947, and (**E**) MJ4. Statistical significance was determined by paired t-test. Open symbols represent female donors and closed symbols male donors.

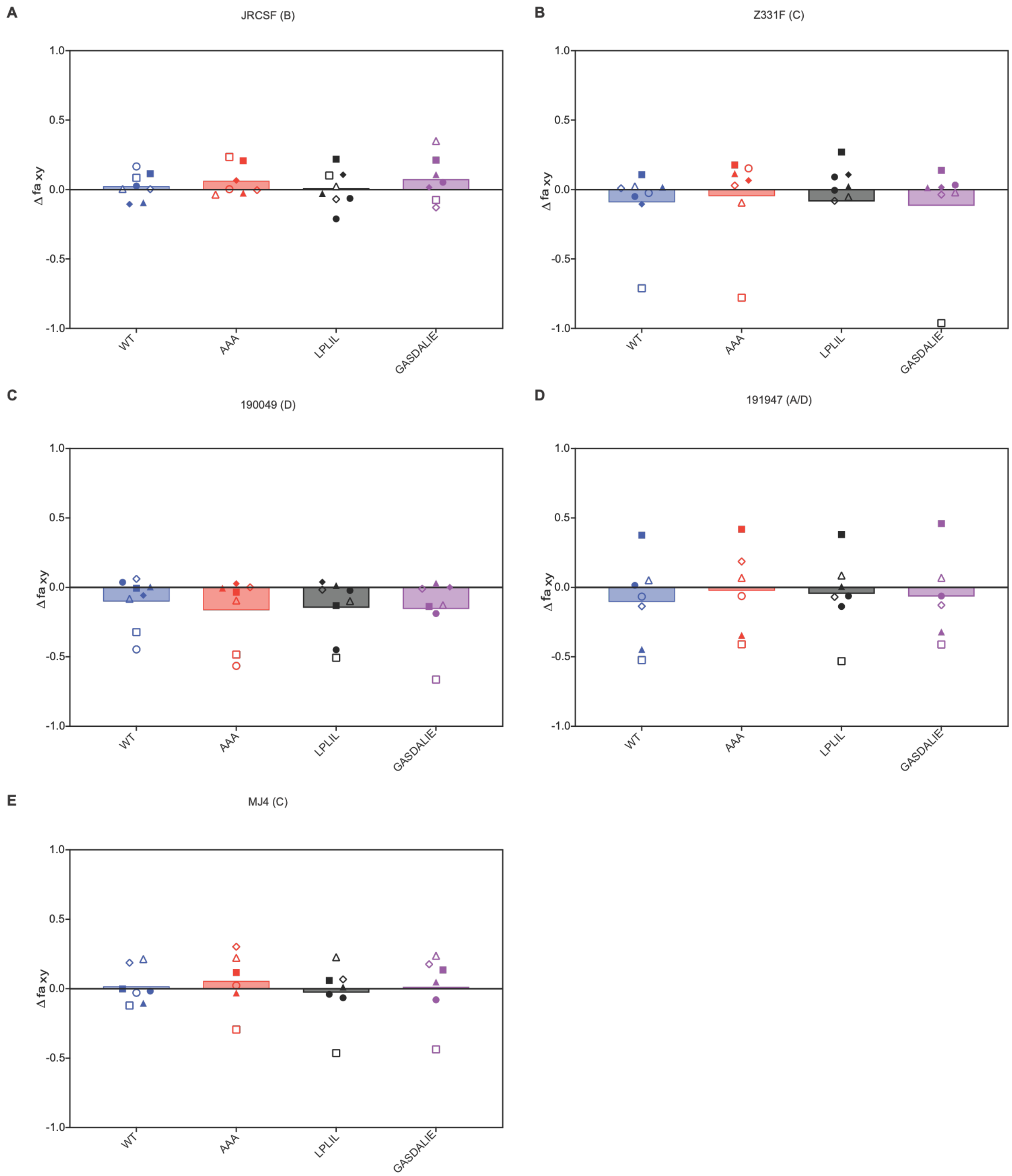

Fig. S15. Synergy evaluation between cytokine treatment and the different Fc-optimized antibodies for experiments shown in Figure 4 and 6. Statistical significance was determined using a one-sample t-test and the Wilcoxon signed-rank test, with a hypothetical value of 0.
